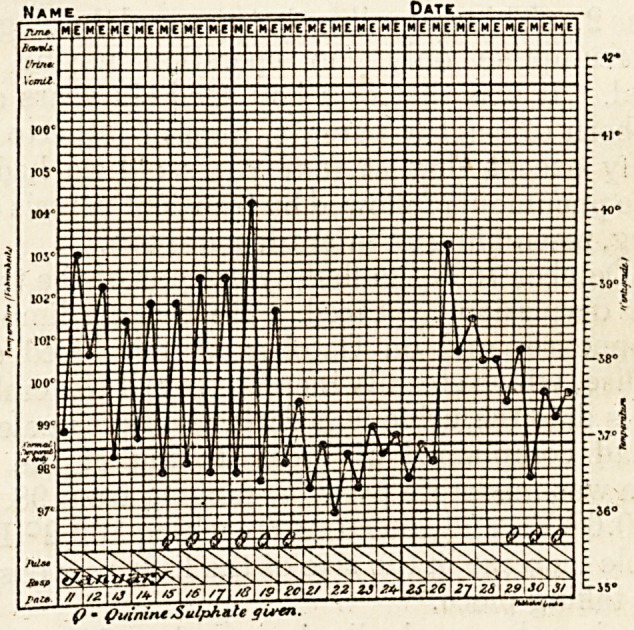# Cholangitis without Jaundice

**Published:** 1908-09-26

**Authors:** 


### SPECIAL ARTICLE.

### CHOLANGITIS WITHOUT JAUNDICE.

It is well known that suppurative pylephlebitis may go on to the formation of multitudes of small abscesses in the liver without there necessarily being any jaundice; and the fact is not surprising, seeing that the branches of the portal veins may be much inflamed or even surrounded by purulent infiltration without the bile ducts within the liver being obstructed to occlusion. Moreover, there may be purulent inflammation of the bile canaliculi themselves without jaundice, or at least without more than a transient and insignificant jaundice. It is true that suppurative cholangitis is associated with considerable jaundice far more often than not; but the absence of persisting jaundice does not exclude the diagnosis of suppurative cholangitis.

The two cases that we propose to give not only illustrate this point, but they also serve as examples to show that infective ascending cholangitis may arise spontaneously, that is to say, without definite antecedent cause, such as gall-stone or growth of the gall-bladder; that it may be due to the bacillus coli communis; that it may be mild in type and comparable to prolonged catarrhal jaundice as regards evolution and the greater part of the symptoms, with the exception of the jaundice itself. In other words, it may be clinically a variety of prolonged catarrhal jaundice in which the jaundice rapidly disappears though the other symptoms persist; whilst at the other end of a continuous chain of cases there are those in which the symptoms, the pyrexia, the rigors, and so forth are so severe—albeit jaundice may still be absent or merely transient—that opening and draining the gall-bladder seems to be the correct treatment, although there is no sign of lithiasis, and although the bile may appear natural.

In all the cases, mild and severe, urotropine in full doses is likely to be of great assistance in treatment, for the reasons that have been already pointed out elsewhere; and bacillus coli vaccine would probably be beneficial also. The cases illustrative of these points are two, recorded by Professor Gilbert and Dr. Lereboullet in Paris.*

*Case* 1.—A lady, aged twenty-seven, entered a hospital on March 22, 1900, for an exacerbation of a febrile illness from which she had suffered for nearly five months previously. The pyrexia was of the quotidian remittent type, and it was accompanied by pains in the hepatic region. Quinine was tried, in case the trouble might be malarial; this is discussed again below. The patient had usually been in good health, except that she suffered from habitual constipation, and that her motions were often mixed with membrane mucus, or blood and mucus, apparently from a muco-membranous entero-colitis.

In February 1899, without any preceding digestive disturbance, and without any known chill or other cause, slight but definite jaundice had appeared, appreciable both by the patient and by her friends, with yellow tinting of the conjunctivæ, pallidity of the fæces, and darkening of the urine. There was no pain, and no pyrexia. Her doctor diagnosed simple catarrhal jaundice, and noted a slight enlargement of her liver. The jaundice lasted about three months, and then the fæces recovered their normal colour and the urine became natural again, the icteric tint of the skin disappearing gradually.

The patient remained in apparently good health from May to August 1899, when transient attacks of pain began in the upper part of the abdomen on the right side and radiated towards the epigastrium. This was accompanied by abdominal distension and constipation, but not by vomiting. Her doctor found the liver still uniformly and slightly enlarged, and the gall-bladder was palpable. A course at Vichy was advised.

A week after she started the Vichy cure she left and went to join her family in the country. The day after her arrival she was seized with the most violent pains in the hepatic region, accompanied by vomiting and abdominal distension. The liver was found to be considerably enlarged, reaching three fingers' breadth below the costal margin. There was pyrexia with rigors. The stools were pale, the urine dark, and there was a slight icteric tint of the skin and conjunctivæ. A week later the attacks of pain and vomiting were still recurring, with unabated severity, the temperature was rising to over 104° F., and there were severe rigors from time to time. The liver was now found to be enormous, extending down as far as the iliac fossa, uniform, not very firm, and slightly tender. At this time there was a suspicion that there might be a hepatic abscess, and that this had burst or leaked into the peritoneal cavity. A week later, however, all the symptoms had greatly ameliorated, and a fortnight later still the vomiting and pain had

Q = Quinine Sulphate given.

ceased entirely, and the liver had returned to its normal dimensions.

The patient remained well until the beginning of November 1899. She then began to suffer once more from rigors and pyrexia. The fever had the intermittent hepatic type; about four or five in the afternoon there was an intense rigor, followed by violent fever, the latter falling again during the night with profuse perspirations. After a month of this, further consultations were held. The liver was enlarged

Q = Quinine Sulphate given.

once more, now reaching several inches below the costal margin; there was no concomitant enlargement of the spleen, nor any other abdominal abnormality. There was no jaundice whatever now, but infective cholangitis was diagnosed. Quinine sulphate was given in doses varying from 5 to 10 grains, and after six days the temperature fell to normal for several days. It rose again not long after, however, seemed once more to be influenced by quinine sulphate, and then became persistent whether quinine was given or not.

For nearly a month between the periods at which the above two portions of temperature charts were taken the patient remained comparatively well; but

* "Bulletins et Mém. de la Soc. Méd. des Hôp. de Paris," ser. iii. vol. xvii. p. 477.

towards the end of February 1900 the urine was found to contain albumen for the first time. In March the rigors, the pyrexial attacks, and sweats returned; there was still no jaundice, but the liver again became enlarged. Quinine had much less influence upon the pyrexia than before. The patient, though still relatively well covered, was losing ground, and it became clear that something operative was required if life was to be saved. Not only was there no jaundice, but even the urine contained so little bile pigment that it was only detected on careful examination, and this not every day. About the end of March laparotomy was performed over the region of the gall-bladder. The latter was small, and exhibited no obvious signs of inflammation, though it contained two small calculi. The liver was greatly enlarged, smooth, congested, and free from all sign either of cirrhosis or of deformity, such as might result from a big abscess growth, or a hydatid cyst. The gall-bladder was drained. The bile obtained from it at the operation, though normal in general appearance, yielded a pure growth of the *bacillus coli communis*. The same organism was obtained from the bile on several subsequent occasions; and also twice from the urine, indicating that the albuminuria was due to an actual "biliary nephritis" arising from organisms that had escaped past the liver into the general circulation.

The patient's recovery was very gradual and slow. The operation did not bring about any sudden amelioration in her condition; but that it was a prime factor in her recovery was shown by the fact that when the biliary fistula became temporarily closed on one occasion for over a week, there was a return of the rigors and perspirations, which rapidly subsided again when the fistula was once more laid thoroughly open. The liver became small again as the patient got well.

*Case* 2.—This was milder in type, and the diagnosis is presumptive only, no operation having been needed. It was that of a boy aged six years and a half, born of healthy parents, himself hitherto quite healthy except that at four years old he had had double pneumonia followed by transient paresis of his left leg, resembling an infantile paralysis.

In October 1899 the first symptoms of the trouble under discussion appeared. He became limp, lost his appetite, and on October 15 developed slight jaundice, which steadily increased until November 1. At this date the urine was dark, the fæces pale, and the lad complained of great itching of the skin. There was moderate pyrexia, ranging from 98.6° F. to 100.6° F., and sometimes reaching 102.2° F. A notable increase in the size of the liver was observed to be taking place.

The jaundice gradually decreased, and it was quite gone in the early part of December. Notwithstanding this, however, the other symptoms persisted, especially the slight pyrexia and the severe pruritus. Epistaxis occurred several times. On December 29, since progress seemed to be unsatisfactory, the lad was taken to the hospital. There was now no jaundice whatever; the urine and fæces were of natural colour; the liver was very large and tender; the spleen could not be felt; all the other organs seemed natural; cholelithiasis seemed improbable owing to the patient's age; and a diagnosis of infective cholangitis without jaundice was made.

No great change one way or the other occurred during January and February, 1900. Jaundice was persistently absent, but pruritus continued very troublesome, and it was accompanied by fleeting eruptions of an urticarial nature. Appetite was very poor, constipation troublesome, and vomiting frequent. There were obscure pains in the hepatic region, extending through to the back and upwards behind. The temperature was of the quotidian type, but not infrequently it was of the reversed type.

A blood count showed 19,530 leucocytes per cubic mm., but the polymorphonuclear cells were no more than 66 per cent. in the differential count. There seemed to be no reason for adopting operative measures unless the condition became worse, as it did in the first case; and slowly but surely the boy progressed towards recovery. Nothing arose to lead to any alteration in the diagnosis. The urine in this case remained quite free from albumen.

To summarise: Case 1 was that of a lady who had suffered from muco-membranous entero-colitis for a long time, and then developed a biliary infection that was at first slight, and included the production of a slight jaundice of the simple catarrhal type; then probably as a result of this infection, two small biliary calculi formed and led to an increase in and spread of the infective trouble, with consequent exacerbation of the symptoms three months later; the biliary infection became progressively more and more severe, although *without any trace of jaundice*, intermittent hepatic fever, variable hypertrophy of the liver, rigors, and attacks of night-sweating being the main symptoms; the kidneys becoming infected finally also, with consequent albuminuria.

In Case 2 the original slight catarrhal jaundice entirely disappeared, but the biliary infection still showed itself in the continuance of pyrexia, hepatic tenderness, and hypertrophy of the liver without concomitant enlargement of the spleen; the course being slowly in the direction of natural resolution.

In each case cure might probably have been accelerated by the use of two modern methods of treatment—namely, full doses of urotropine, and a bacillus coli vaccine hypodermically.

The most noteworthy point in the two cases is the absence of jaundice when the illness was at its worst. After an initial phase of slight catarrhal jaundice, this disappeared entirely. There was neither cholæmia nor choluria. It would seem that acute infective cholangitis may produce its severe symptoms without entirely obstructing the finer bile ducts. Bile reached the intestine, as was shown by the coloration of the fæces after the earlier stages of the illness were past.

Pyrexia with rigors is one of the capital symptoms of infective cholangitis; and it is worthy of note that the temperature may be influenced to some extent by quinine, so that there may be a difficulty in excluding malaria. The latter was at one time suspected in the first case, but it was excluded both by blood examinations and by the subsequent course of the disease. The two cases exhibit two very different degrees of severity of the same disease; doubtless there are cases that are milder even than case 2; probably there are examples of greater severity than case 1; and it is certain that cases will be met with presenting every intermediate degree of severity between the two.

## Figures and Tables

**Figure f1:**
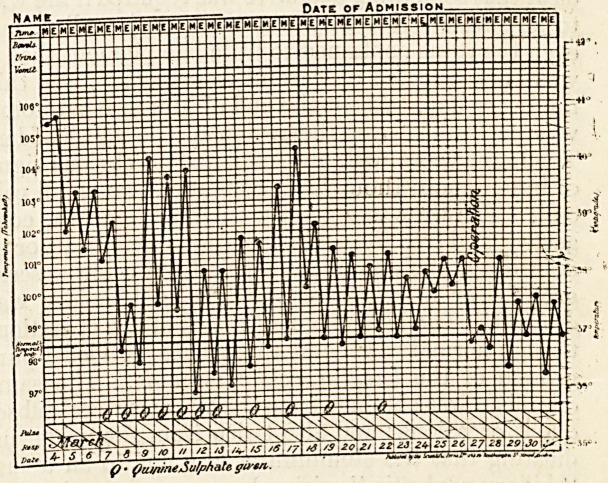


**Figure f2:**